# Gastrointestinal Duplications: Experience in Seven Children and a Review of the Literature

**DOI:** 10.4103/1319-3767.61237

**Published:** 2010-04

**Authors:** Abdur-Rahman L. Olajide, Abdulkadir A. Yisau, Nasir A. Abdulraseed, Ibrahim O.O. Kashim, Adeniran J. Olaniyi, Adesiyun O.A. Morohunfade

**Affiliations:** Pediatrics Surgery Unit of Department of Surgery, University of Ilorin Teaching Hospital, Ilorin, Kwara State, Nigeria; 1 Department of Radiology, University of Ilorin Teaching Hospital, Ilorin, Kwara State, Nigeria; 2 Department of Pathology, University of Ilorin Teaching Hospital, Ilorin, Kwara State, Nigeria

**Keywords:** Bowel gangrene, children, enteric duplication, surgery, volvulus

## Abstract

**Background/Aim:**

Enteric duplication (ED) is a rare congenital anomaly that can occur anywhere along the alimentary tract from the mouth, down to the anus and the nearby organs. This uncommon anomaly may be asymptomatic or presents with vague symptoms mimicking other common pathologies. We aim to present our experience, management challenges and patterns of ED with a review of the literature.

**Settings and Design:**

The study was carried out at a Nigerian Tertiary Hospital (2005–2008 inclusive).

**Materials and Methods:**

We retrospectively analyzed seven patients with ED managed in our hospital for sex, age, clinical presentations, duplication size and site, presence of ectopic tissue, complications, associated anomalies, radiological workups, and prognosis. Data was analyzed using SPSS 11.0 for window.

**Results:**

Seven children between the age range of 44 hours–10 years had ED, one sublingual and six intraabdominal duplications. Midgut volvulus with long segment bowel gangrene complicated two cases. The diagnosis was incidental in all. Three cases were diagnosed following conventional radiological contrast examination and the rest at surgery. Ultrasound was not helpful in making diagnosis in all the six intraabdominal duplications. Though surgery was recommended for all, one of the patients declined. Only one patient had unsuccessful surgery.

**Conclusions:**

ED requires high index of clinical suspicion and careful management. Many cases of nonspecific abdominal pains should be properly evaluated before patients suffer avoidable complications.

Enteric duplication (ED) is a rare congenital anomaly that can occur anywhere along the length of the alimentary tract from the mouth down to the anus and the nearby organs.[1–7] Overall, ileum is the most common site of occurrence.[1–7]

The pathogenesis is still vaguely understood. However, errors in normal embryologic canalization or embryologic connection between the developing gut and neural tube, as a part of the split notochord syndrome have been postulated. The diagnosis of an ED cyst is difficult to make clinically because the wide spectrum of symptoms and unspecific signs frequently simulate other diseases.[3,4] The clinical presentations may be vague, diverse, and varied depending on their location.[5] These include nausea, vomiting, bleeding, pain, swelling, distention, dysphagia, dyspepsia, respiratory distress, chronic constipation, and complications including bleeding, perforation, malignancy, and obstruction of the alimentary tract and vessels.[1,2,4–16] Hence, most cases of ED are diagnosed incidentally, especially, at surgery.

Plain X-rays are of limited use in the diagnosis of ED.Conventional contrast radiographic examinations such as swallow, meal, follow through and enemas could be of value if the ED is tubular. Ultrasound and magnetic resonance imaging (MRI) findings may be diagnostic. Computerized tomography (CT) is useful in delineating surrounding structures.[9] Nuclear scan may have a role in the detection of bleeding ED although, it may be unable to distinguish it from Meckel's diverticulum except when other features are identified with concomitant investigations.[10] Once the diagnosis is established, surgical correction is the treatment of choice, preferably complete excision.[9] The outcome is generally good and mortality did not exceed 20% in any series.

We present our experiences with EDs in seven children between 2005 and 2008 and review the literature.

## MATERIALS AND METHODS

We retrospectively analyzed sex, age, clinical presentations, duplication size and site, presence of ectopic tissue, complications, additional associated anomalies and prognosis in seven patients with ED managed in our hospital (2005–2008 inclusive). Radiological work-up of each patient was also analyzed. This is to determine the patterns and the management challenges in these patients.

## RESULTS

The patients’ age range varied between 44 hours–10 years at presentation. Five patients were male. There were eight ED, four (50%) of which were ileal duplication [Table 1]. Abdominal swelling and vomiting, a characteristic feature of intestinal obstruction was the most common presentation. The duplication types, age at presentation, treatment and outcome are as shown in Table 1. Two cases were complicated by volvulus and bowel gangrene. These two patients presented earlier (within 10 days of life) and were the only patients that presented with bilious vomiting [Table 1]. The first was a 44-hour-old infant female neonate with bilious vomiting and progressive abdominal distension. She was delivered by emergency caesarean section on account of preeclampsia in the multiparous mother with no history of polyhydramnios. She passed meconium within six hours of birth but had hyperkalemia (5.9 mmol/L) in spite of adequate urine output and features of small intestinal obstruction on abdominal X-rays. Findings at surgery were turbid purulent fluid with meconium in segment of gangrenous twisted ileum around a cystic duplication. About 64-cm of gangrenous bowel was resected and primary bowel anastomosis done. Baby had a turbulent postoperative period from sepsis and jaundice but was managed successfully and discharged on the 19th day postoperatively. The second case having small bowel duplication with gangrene was 10-days-old at presentation. He had done well in the immediate postnatal period. A few hours after presentation, he developed sudden abdominal swelling, fever, progressively irritable, and vomited several times. Clinical features were in keeping with intestinal obstruction suspected to be malrotation. However, laparotomy confirmed cystic duplication of the midgut twisted around itself. Resection and anastomosis surgery were performed and were uneventful.

**Table 1:** Demographic and clinical patterns of enteric duplications

**Cases**	**Age**	**Sex**	**Duplication**		**Presentation**	**Treatment**	**Outcome**	**Other findings**
							
			**Sites**	**Types**				
1	4 year	Male	Sublingual 5×6 cm^2^	Cystic	Sublingual/cervical swelling, drooling	Excision	Satisfactory discharged 5-DPO	Nil
2	10 day	Female	Midgut (40 cm long)	Cystic	Abdominal swelling, bilious vomiting	Resection and jejunoileal anastomosis	Satisfactory discharged 12-DPO	Gangrenous bowel and mid-gut volvulous
3	44 hours	Female	Ileal (64 cm long)	Cystic	Abdominal swelling bilious vomiting	Resection and jejunoileal anastomosis	Satisfactory discharged 19-DPO	Gangrenous bowel and ileal Volvulous
4	7 year	Male	Descending colon (14 cm long)	Cystic	Abdominal pain and distension	Resection and coliocolic anastomosis	Satisfactory discharged 9-DPO	Nil
5	4-month	Male	Terminal ileum, caecum, colon and appendix	Tubular	Recurrent constipation and failure to thrive	Ileostomy because of bad state	Died 3wks later from sepsis and malnutrition	True duplication
6	2-month	Male	Sigmoid colon	Tubular	Recurrent abdominal swelling	Parent decline surgery	-	-
7	3-month	Male	Ileal (12 cm long)	Cystic	Abdominal pain and swelling	Resection and ileo-ileal anastomosis	Satisfactory discharged 9-DPO	Nil

Four duplications demonstrated ectopic gastric mucosa while the rest showed mucosal pattern of the adjacent gut most closely related. One parent declined surgery because of fear and lack of funds. Excision surgery and anastomosis gave satisfactory results in all patients, but one died of sepsis and malnutrition three weeks after the surgery.

## DISCUSSION

ED, an uncommon malformation of the gastrointestinal tract, may be asymptomatic or presents with vague symptoms mimicking other more common pathologies such as intussusception, volvulus, appendicitis, pelvic abscess, diverticulitis, achalasia, and Hirschsprung's disease.[1,2,4–17] It is most commonly diagnosed when complications such as bleeding, intestinal obstruction or perforation occurred.[5–10] Multiple theories have been proposed to account for ED but no single theory adequately explains all the known duplications. Majority of ED are single, cystic and located on the mesenteric side of the native alimentary tract. All the intraabdominal ED in this study were located at the mesenteric edge of the bowel.[4–9] Symptoms are often related to the location of the duplication. Oral and esophageal lesions may cause respiratory difficulties, whereas lower gastrointestinal lesions may cause nausea, vomiting, bleeding, perforation, or obstruction.[4–9,13,14]

**Figure 1:**
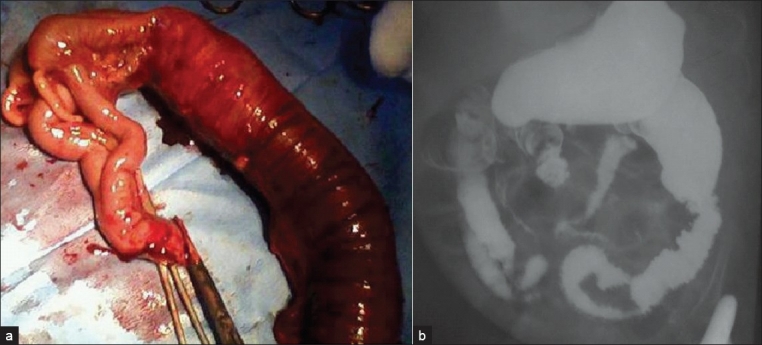
(a) Resected bowel of a four-month-old child (Case 5) showing duplicated terminal ileum, caecum appendix and distal colon. (b) Single contrast barium enema of same patient. Note the duplicated bowel, and dilated transverse colon

All ED presented in this series had obstructive gastrointestinal symptoms with exception of one who had sublingual cystic duplication. This patient presented with drooling of saliva but had no dysphagia or respiratory difficulty despite the size being approximately 5×6 cm^2^. According to Chen *et al.*[14] who reported two cases of sublingual duplication cysts, intraoral ED cyst is rare and has the potential for airway obstruction and respiratory distress at delivery that may necessitate immediate tracheostomy where it presents as a large sublingual mass. Unlike in the Chen *et al*.[14] cases, where antenatal diagnosis with ultrasound allowed for the proper preparation of personnel and equipment in the management of those neonates during delivery even before clamping the umbilical cords, our patient with sublingual cyst did not present until four years of age but still complete cyst excision [Figure 1] was possible.

Approximately 75% of ED are intraabdominal and over half of which are ileal duplications. The findings of four ileal, three colonic, and a sublingual ED in our series is consistent with the pattern described previously. The frequency of cystic ED in our series (five patients) was similar to 75% of cystic duplications reported by some authors.[1–5] The spherical or tubular forms of duplication, which occurred in two of our patients, had colonic involvement. Six of our patients (71.4%) were symptomatic in the first year of life, which is similar to over 60% reported by several authors.[1–5]

Some ED may not be symptomatic until at school age or adulthood.[1–5] Therefore, the diagnosis of a duplication cyst is difficult to make clinically or based on conventional radiologic study including barium examinations (swallow, meal, follow through, and enemas) because of the wide spectrum of symptoms and the unspecific signs that may frequently simulate other diseases.[3,6,7,9] None of our case was suspected clinically and this created a challenge intraoperatively. Nevertheless, two patients had positive barium enema findings, one of which had tubular duplication of both ileum and colon. Again, two of our patients suspected to have Hirschsprung's disease clinically, had their cystic duplications complicated by volvulus and gangrene. Only these two patients presented with bilious vomiting. One of them died three weeks postsurgery.

Heterotopic mucosa of gastric and pancreatic origin is a common finding in ED. Ectopic gastric mucosa was seen in four of our patients. Noteworthy complications such as gastrointestinal ulceration and hemorrhage from ectopic gastric mucosa, bowel perforation and peritonitis, malignant degeneration and intussusceptions have been documented.[8–12,15–17] There could be more than one type of heterotopic mucosa in the same duplication. When acid secreting mucosa lines the lesion, hemorrhage or erosion may result. None of these occurred in our patients that demonstrated ectopic gastric mucosa. Although additional malformations (of the genitourinary or vertebra) have been encountered in 16–26% of the cases in some series,[4] there was none in our series.

Within this series, volvulus complicated by long segment bowel loop gangrene of 64 cm and 45 cm occurred in two patients, respectively. Thus, the signs and symptoms leading to diagnosis varied between duplications, the age of patient, location of the duplication, type of mucosal lining, duration of disease, and presence of complication.[4]

The diagnosis is rarely made until at surgery because of nonspecificity of symptoms and presentations. Radiological work up with ultrasound, computed tomography (CT), and magnetic resonance (MRI) have been useful. MRI has capability of defining synchronous cyst and spinal cord anomaly but is relatively expensive and scarce.[1,3,4] Where duplication is tubular, barium examination if not contraindicated may be diagnostic. Both our patients with tubular duplications were thought to have Hirschsprung's disease on clinical evaluation but barium enema examinations done on them were consistent with ED.

Plain thoracic and abdominal x-rays and ultrasonography are the most commonly used diagnostic radiological methods in our center when gastrointestinal obstructive symptom is present. The plain radiographic features were nonspecific in all our patients but were capable of excluding vertebral anomaly. The diagnoses were missed on abdominal ultrasonography probably due to low index of suspicion and limitation imposed by overlying bowel gas because all our patients with intraabdominal ED presented with gastrointestinal obstructive symptoms.

The ultrasonographic and MRI feature suggestive of duplication is identification of a three-layered image representing the duplication cyst, common wall, and outer bowel wall.[1,3,4] These imaging modalities have assisted in prenatal diagnoses in some cases. In many developing countries, this opportunity may remain elusive over time as many mothers still practice home delivery because of ignorance and poverty, let aside the scarcity of MRI and its cost. The role of multimodality imaging cannot be over-emphasized. Where ED is associated with bleeding, availability of isotopic scan facility, with material such as Technetium 99m pertechnetate, could be useful in demonstrating the bleeding mucosa from the ectopic tissue, as demonstrated for Meckel's diverticulum.[10]

There have been some cases of mistaken diagnosis leading to wrong management of patients and attendant complication such as persistent perineal fistula, which occurred in two patients thought to have pelvic abscess and drainage instituted in a series reviewed by Iyer *et al*.[9]

Treatment of EDs is by surgical means. The main considerations in the management of ED are the age and condition of the patient, location of the lesion, whether it was cystic or tubular and communicating with the true intestinal lumen, and whether it involves one or more anatomic locations. Generally, total excision is preferred, but staged approaches are sometimes necessary.[8,14,18] Sacrificing a segment of normal intestinal tract is sometimes necessary during the resection of ED because of the often-intimate attachment, but one should avoid long segment resection which may result in a short bowel syndrome, though this may be inevitable in cases of gangrenous bowel. Tubular duplications rarely have an autonomous blood supply and since it has an 80–100% incidence of gastric mucosa, simple drainage of the distal end may cause peptic ulceration of the normal mucosa with bleeding or perforation, and thus should be discouraged. The Wrenn method of mucosa stripping can be used even for long tubular duplication, although multiple incisions like a step ladder may be necessary.[19] There have been some cases, though very few, treated by laparoscopic-assisted resection.[17–22] Overall, laparotomy is still commonly performed anyway. The results of surgery are generally favorable, with not more than 20% mortality in any reported series to the best of our knowledge. However, some cases of ED have been reported to have developed malignancy.[11,12]

## CONCLUSION

ED exists in our environment and demands a high index of suspicion and careful clinical management. Many cases of nonspecific abdominal pains should be properly reviewed and investigated before patients suffer avoidable complications.
